# Correspondence

**Published:** 1912-05

**Authors:** 


### Correspondence.

CHICAGO, April 9, 1912.

*Dr. F. E. Daniel, Austin, Texas.*

DEAR DOCTOR: I have just read with interest your paper in the TEXAS MEDICAL JOURNAL on "The Castration of Sexual Perverts." It is great. Just as soon as I get the time to study that question a little, I am going to take it up and do what I can to second your endeavors in this matter. It is the right thing, doctor, and I believe that if punishment is intended to deter from crime rather than for any other purpose that this will have a greater influence than any other penalty which could be inflicted on man.

Cordially yours,

W. F. WAUGH,
Dean, Bennett Medical College.

---

JOHNSON CITY, TEXAS, April 5, 1912.

*Editor Texas Medical Journal, Austin, Texas:*

It was indeed a pleasure to read your article in the April JOURNAL regarding the treatment of moral perverts. I heartily endorse practically all of your views upon this subject, but my opinion is that we should go further and begin at both ends, not of the human subject, but of the program of prevention of moral lapses in the human subject. By this I mean that we should not only prevent propagation in the individuals you mention—the habitual criminal, drunkard, syphilitic, and mentally unfit—but we should also institute a campaign of education of the youth of this country in moral hygiene as a means of prevention of the acquisition of those habits, the inevitable sequence of which is the perpetration of criminal acts.

I believe there is a way to accomplish this, and, though some more feasible way may be advocated by others, at the present time I advocate the plan of beginning with our youth, as follows:

In every community some physician or physicians should be delegated to give a series of lectures in every schoolhouse to pupils of certified age on moral hygiene, the importance of the use and abuse of the sexual organs, together with the disastrous results of acquiring disease by indulging in illicit intercourse, and the weakening of the mental faculties which follows such practice as self-abuse.

The plan above outlined would include other subjects in line with the general object of bringing before our boys and girls the importance of morality in their everyday life.

This plan should be followed for the space of two years, in which time a chair of moral hygiene should be founded in our University, in order to prepare the future teachers in our schools to take up this work.

There is no doubt that the physicians of the land are at the present time the only class capable of doing this work, and until our teachers are so prepared it is up to them to start the campaign, and they should be only too willing to do it for the good of our youth.

This plan I advocated in one of our district meetings, but some of my colleagues thought it smacked of advertising. Really, I do not think it does so any more than warning the public against tuberculosis or any other disease, which we are doing by means of public talks.

Another objection to this plan was that we should not do this in public but in the home of the children and amongst the parents. This has been done in a great measure, yet the present state of this question is deplorable, notwithstanding these efforts.

Summing up this plan, it amounts to this:

First. Let the physicians of our State take such interest in the subject that they will help for a time to educate the youth.

Second. Let a chair of moral hygiene be founded in our University for the preparation of future teachers in this important subject.

Third. Let every teacher attend such lectures as are given by the occupant of this chair, and so prepare himself or herself as to be able to intellectually bring before their pupils the importance of this suject and in time take this duty off the shoulders of the physician.

In this way we begin at the beginning, or rather at the foundation of this matter, and in time to come we will not see so many subjects of moral perversion who require an operation which is offensive to mankind in general.

Yours fraternally,

GEO. HARWOOD, M. D.

